# Expressional and prognostic value of TREM1 in ovarian cancer: A comprehensive study based on bioinformatics and clinical analysis validation

**DOI:** 10.7150/jca.101152

**Published:** 2025-01-01

**Authors:** Caihong Tang, Dong Ye, Qiong He, Qionghua He, Wenkai Zhou, Liya Lin, Chao Jiang, Da Huang, Jianwei Zhou

**Affiliations:** 1 Department of Urology, the Second Affiliated Hospital, School of Medicine, Zhejiang University, Zhejiang, China.; 2 Department of Urology, Longquan People's Hospital, Zhejiang, China.; 3 Department of Gynecology, the Second Affiliated Hospital, School of Medicine, Zhejiang University, Zhejiang, China.; 4 Department of Anorectal, Shengzhou Traditional Chinese Medicine Hospital, Zhejiang, China.; 5 Laboratory Animal Research Center, Academy of Chinese Medical Sciences, Zhejiang Chinese Medical University, Zhejiang, China.

**Keywords:** TREM1, ovarian cancer, prognosis, tumor microenvironment, pan-cancer

## Abstract

**Background:** Triggering receptor expressed in myeloid cells-1 (TREM1) is an important regulator of innate and adaptive immunity, which can directly amplify an inflammatory response. Current studies have found the immunomodulatory role of TREM1 in tumor microenvironment. However, the role of TREM1 in ovarian cancer (OV) remains unclear.

**Methods:** Based on TCGA and GEO database, we performed bioinformatics analysis to evaluate the expression profile of TREM1. Then, the prognostic value of TREM1 was determined through Kaplan-Meier survival analyses. GO and KEGG enrichment along with GSEA analyses were performed to identify potential biological functions of TREM1 based on the gene co-expression network. IHC and RT-qPCR on clinical samples were performed to validate our database-derived results. Additionally, ESTIMATE and CIBERSORT analyses were used to assess the correlation between TREM1 and tumor microenvironment. Finally, the expression, prognosis and immune regulation patterns of TREM1 in pan-cancer were further explored to validate the role of TREM1 as a biomarker.

**Results:** The expression of TREM1 is abnormally high in OV than in normal tissues. Patients with high TREM1 expression were linked with poor overall survival (OS) and disease-free survival (DFS). Then, cox regression analysis and a nomogram indicated that TREM1 was an independent prognostic factor and proved the effective predictive performance in OV. Enrichment analysis showed that TREM1 was highly enriched in cancer-and immune-related pathways. Additionally, immune analysis revealed that TREM1 was robustly positively associated with tumor-associated macrophages (TAMs) and regulatory T cells (Tregs) infiltrating. Moreover, pan-cancer analysis showed TREM1 was closely associated with prognosis and immune-related genes expression in various types of cancer.

**Conclusions:** Through a systematic and comprehensive analysis, our study revealed that TREM1 could serve as a prognostic and immunological biomarker in ovarian cancer.

## Introduction

Despite being the third most common gynecologic malignancy worldwide, ovarian cancer (OV) has the highest mortality rate among those diagnosed. Despite substantial recent advance in the understanding, treatment and management of OV, the overall patient survival rate is still dismal due to very limited early-stage symptoms, high rates of metastasis and recurrence. In fact, above 70% of patients are detected at advanced stages when they first get diagnosed, with a 5-year relative survival rate of only 29%^1^, ^2^. The majority of OV patients experience recurrence after standard therapeutic approaches and the clinical improvements from cancer therapies are typically insufficient due to the highly heterogeneous. The interactions between cancer cells and stromal cells and extracellular components will create a conducive environment for tumor growth, which contribute to cancer progression and resistance to anti-tumor therapy^3^, ^4^. To design more effective therapy methods, it is essential to comprehend the molecular pathways through which the microenvironment of OV contributes to genesis and progression of OV.

The triggering receptor expressed on myeloid cells (TREM) family belongs to immunoglobulin superfamily and have been reported to be involved in varieties of pathological conditions, including infectious, sterile inflammatory diseases, cardiovascular diseases and degenerative conditions^5^, ^6^. Among these family members, two major receptors TREM1 and TREM2 had attracted great attention and extensively studied, they share a similar molecular structure and both interact with the transmembrane protein, DNAX-activation protein 12 (DAP12). Comparing to TREM2 as an important negative regulator of immunity, after association with DAP-12, TREM1 can amplify the inflammatory response through inducing the secretion of pro-inflammatory cytokines and chemokines^7^-^9^. Intriguingly, recent studies reported that TREM1 was abnormally expressed in a variety of tumors and had been determined as a central player in patients' poor outcome^10^. The plasma levels of soluble TREM1(sTREM1) were significantly elevated in patients with renal cell carcinoma, hepatocellular carcinoma and non-small cell lung cancer^11^-^13^. Meanwhile, higher plasma concentrations of sTREM1 predicted shorter survival in NSCLC patients^14^. Mechanistically, hypoxia, persistent DNA damage and oncogenic stress and epigenetic changes could upregulate the expression level of TREM1^15^-^17^. Abnormal upregulation of TREM1 could promote the proliferative capacity of cancer cells by secreting various growth-promoting signals and inflammatory cytokines^18^. Furthermore, TREM1 signaling promoted immunosuppressive cell infiltration in tumor microenvironment(TME), such as myeloid-derived suppressor cells (MDSCs), Foxp3^+^ regulatory T cells (Tregs) and tumor-associated macrophages (TAMs) , thereby impacting the anti-tumor activity of CD8+T cells^17^. However, other studies showed opposite results. An agonistic TREM1 monoclonal antibody (PY159) could upregulate the secretion of inflammatory factors such as IFN-γ, CCL3, CCL4, IL-8, and TNF and increased tumor sensitivity to anti-PD-1 therapy in syngeneic mouse tumor models^19^. Overall, the differences in the results of these studies prompted us to investigate the interaction between TREM1 in ovarian tumors and the immune microenvironment.

In this study, we firstly aimed to explore TREM1 expression patterns in ovarian tumor as well as their association with prognosis of survival. Our tissue microarray data validated that high expression TREM1 was unfavorable overall survival of OV patients. In addition, we found that TREM1 expression was strongly linked with tumor-infiltrating immunological cells using a series of bioinformatics data from public platforms. Therefore, TREM1 could be a promising biomarker and novel therapeutic target for ovarian tumors.

## Materials and Methods

### Data collection

TREM1 expression levels and patients' clinical data in TCGA-OV (including 425 OV patients) were obtained from UCSC Xena (https://xena.ucsc.edu/). Similarly, GSE18520 dataset (including 53 OV patients and 10 normal patients) were also taken from GEO dataset(https://www.ncbi.nlm.nih.gov/geo/query/acc.cgi?acc=GSE18520).

### Gene mutation and expression difference analysis

The CBIoPortal (http://www.cbioportal.org) is a free website which facilitates exploration and visualization of multidimensional cancer genomics datasets. GEPIA (http://gepia.cancer-pku.cn/index.html) is a web-based bioinformatics tool for analyzing gene expression via TCGA and GTEx. The association between TREM family members (TREM1, TREM2, TREML1, and TREML2) and gene mutation and expression difference in OV was confirmed using above-mentioned two databases, respectively.

### Survival and prognostic analysis

Correlation of TREM family members' expression between overall survival (OS) of patients from TCGA cohort were used by “survival” and “survminer” R packages. Univariate and multivariate Cox survival were done to screen out independent prognostic factors associated with survival in OV. According to multivariate Cox proportional hazards analysis, a nomogram was designed to forecast 1,3,5-year OS and calibration curves was used to evaluate the goodness-of-fit of the nomogram.

### Tissue microarray and immunohistochemistry (IHC) assay

Human ovarian cancer tissue microarrays including 160 OV tissue samples (including 155 OV tissues and 5 adjacent non-tumor tissues or borderline ovarian tumor) were bought from Shanghai Outdo Biotech (Shanghai, China). IHC staining using rabbit polyclonal anti-TREM1 antibody (1: 3,00, 11791-1-AP, Protentech, China). IHC score was measured by multiplying staining intensity and staining rate. Staining intensity was scored as “0” for no staining, “1” for weak, “2” for moderate, and “3” for strong. Staining rate was classified as “0” for no positive tumor cells, “1” for 1%-25%, “2” for 26%-50%, “3” for 51%-75% and “4” for 76%-100%. Two pathologists independently estimated Immunostaining results. Score of <8 was considered low expression group, whereas samples with scores ≥8 were considered the high expression group in OV tissues.

### Human tissue samples

Human tissue specimens (including 20 OV and 13 normal ovarian tissue specimens) were collected and stored at the Department of Gynecology, the Second Affiliated Hospital. The study was approved by the ethical committee of the Second Affiliated Hospital of Zhejiang University School of Medicine. All patients had signed the informed consents.

### RNA extraction, RT-PCR, and RT-qPCR

Total RNA was isolated from patients with OV by Trizol reagent (Macherey-Nagel, Germany), and reverse transcription was done utilizing PrimeScript™ RT reagent Kit with gDNA Eraser (Takara, Japan). RT-qPCR was done utilizing TB Green® Premix Ex Taq™ II (Takara, Japan) on 7500FAST system (ABI, USA) following the supplier's guidelines. The relative quantification of mRNA expression was represented by 2^-ΔΔCT^.

The primers for TREM1 were: 5'-TGCTCTTTGTCTCAGAACTCCG-3'(forward) and 5'- TGATCCTCCCCACTTGGACT-3'(reverse).

The primers for GAPDH were: 5'-CTGACTTCAACAGCGACACC-3'(forward) and 5'- GTGGTCCAGGGGTCTTACTC-3'(reverse).

### Enrichment analysis of TREM1 gene co-expression network

Firstly, we explored co-expressed genes associated with TREM1 expression in the TCGA-OV datasets in R software. Pearson's correlation coefficient to test the statistical correlation and the “pheatmap” package of R to draw heat map for display. Then, co-expressed genes (*p* < 0.05) and TREM1 were selected for GSEA analysis (including GO, KEGG and Reactome) to investigate the potential regulatory mechanisms. GSEA was implemented by the “clusterProfiler” R package with the following parameters: nPerm = 1000, minGSSize = 10, maxGSSize = 1000, and p-value-Cutoff = 0.05.

### Tumor microenvironment and immunological cell infiltrate analysis

To evaluate the abundance of stromal and immune cells in TME, ESTIMATE algorithm were implemented to calculate the immune and stromal scores through “estimate” R package^20^. Then, CIBERSORT algorithm was utilized to evaluate association between TREM1 level and 26 types of tumor-infiltrating immune cells in OV. In addition, the co-expression analysis of TREM1 and immunological-related genes: genes encoding major histocompatibility complex (MHC), immune suppression, chemokine and chemokine receptors was also analyzed utilizing R-package “limma”.

### Statistical analysis

Data analyses were conducting through R software (version 4.1.0) and GraphPad Prism 9. Kaplan-Meier survival analysis and Cox proportional hazard regression analysis were utilized for prognostic role of TREM1. Pearson correlation analysis was conducted to evaluate the correlation between TREM1 expression and co-expressed genes and immune cell infiltration. The significance difference of the TREM1 mRNA expression levels between tumor and normal tissues was determined by unpaired t-test. For all statistical analyses, *p* < 0.05 was considered statistically significant.

## Results

### Comprehensive analysis of TREM family members

**Figure 1:**
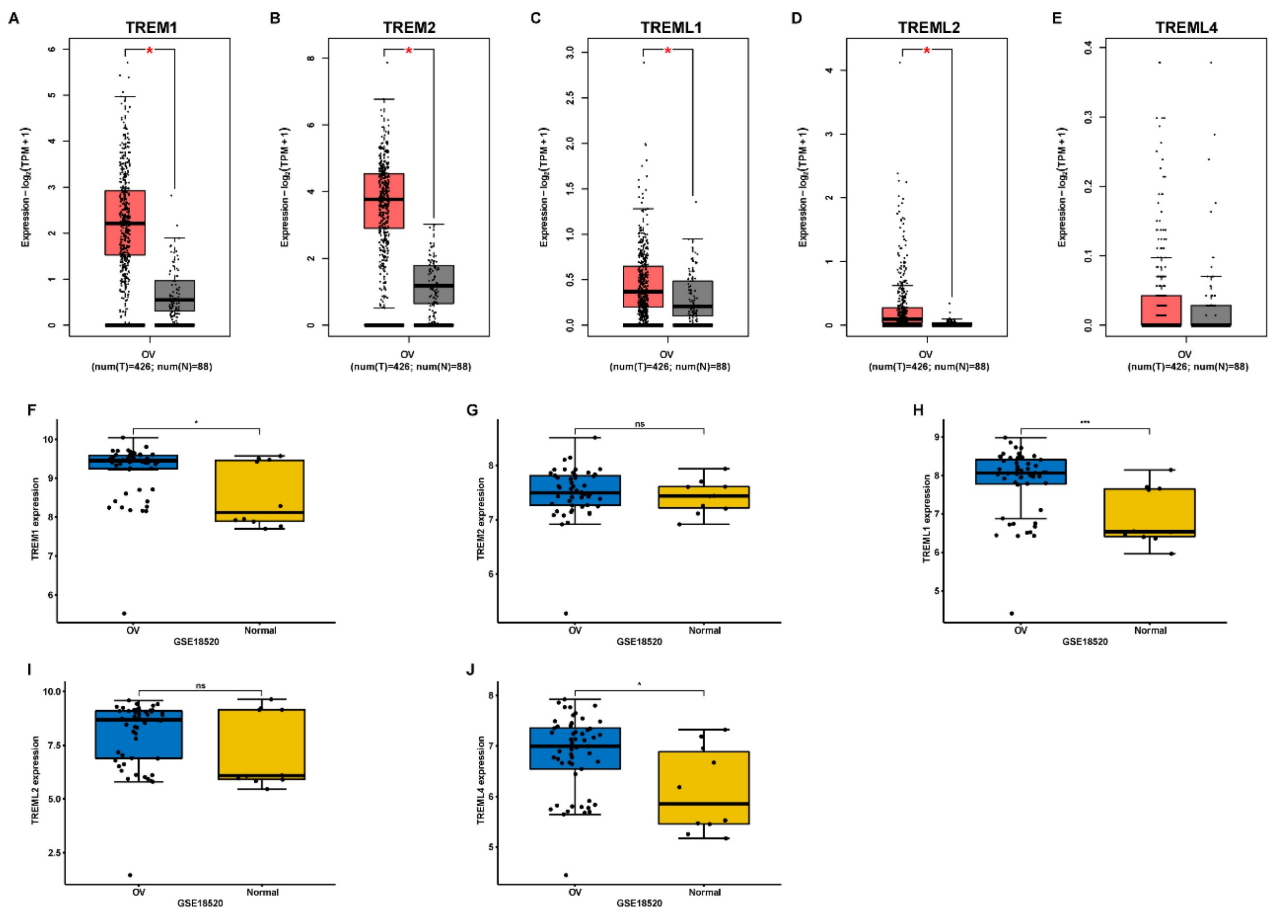
The mRNA expression of TREM family members in ovarian cancer. (A-E) Analysis of the expression of TREMs family members in ovarian cancer tissue compared with normal tissue using GEPIA database. (F-J) Analysis of the expression of TREMs family members in ovarian cancer tissue compared with normal tissue using GEO database. *p < 0.05.

**Figure 2:**
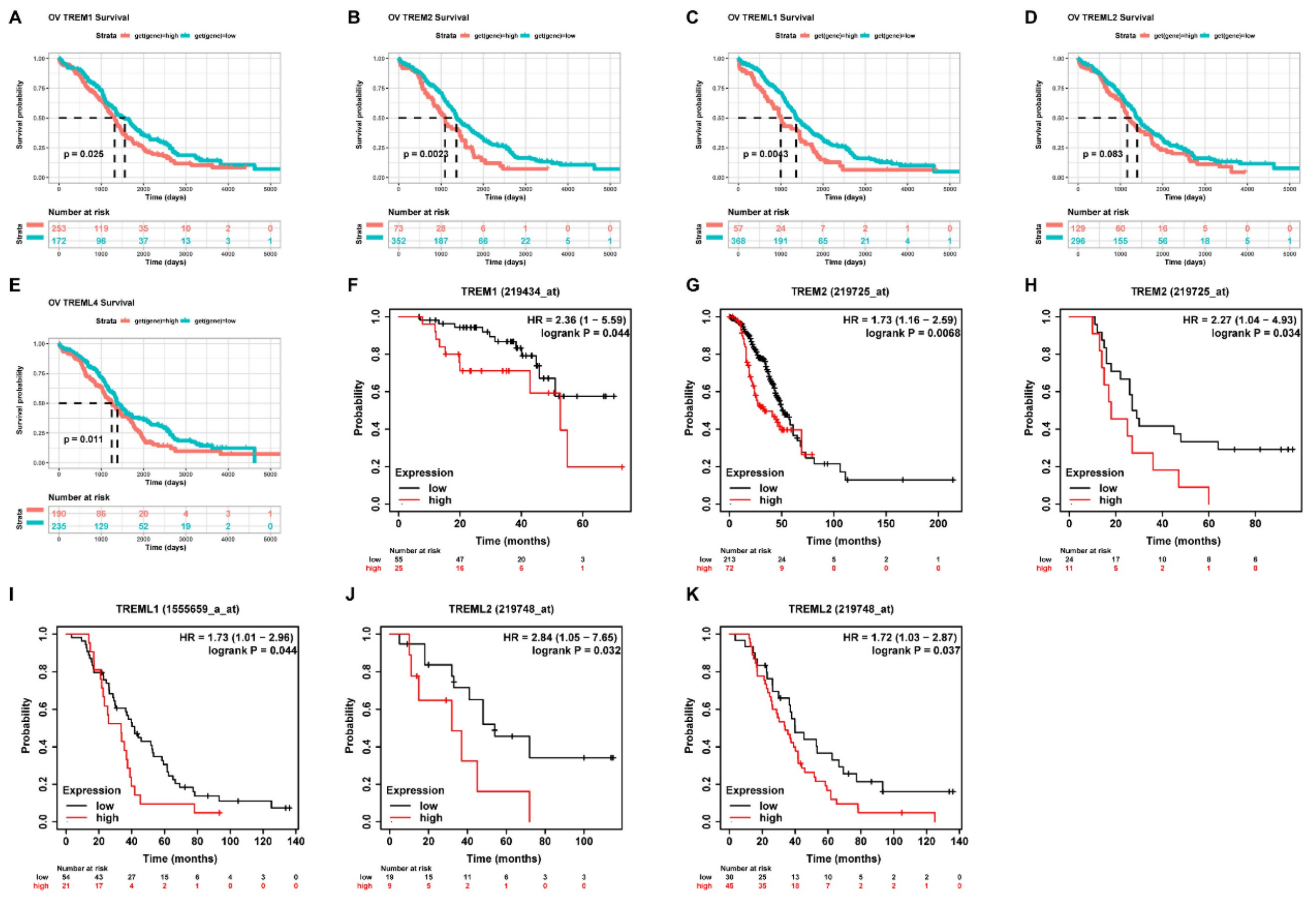
The Correlation analysis between TREM family member expression levels and overall survival. (A-E) Kaplan-Meier analysis of the association between TREM family member expression and overall survival in TCGA cohort. (F-K) Kaplan-Meier analysis of the association between TREM family member expression and overall survival in GEO cohort.

First, using the GEPIA database, we investigated the mRNA expression levels of TREMs family genes among cancer tissues and normal tissues in ovarian cancer (OV). Results showed that the expression of TREM1, TREM2, TREML1, and TREML2 were all found to be significantly overexpressed in OV (n = 426) than in normal tissue samples (n = 88) (Figure 1 (A-E)). The TREM family members' expression levels were also estimated in one GEO datasets (GSE18520) and found that the expression of TREM1, TREML1 and TREML4 were upregulated when compared to the normal tissues (Figure 1 (F-J)). Subsequently, we investigated the interaction between the aforementioned genes at transcriptional and protein levels and revealed that TREM1 and TREM2 had relatively strong correlation (Supplementary Figure 1(A-B)). Representative images of immunohistochemical stains of TREMs family genes (lacking TREML2) in ovarian normal and cancer tissues were acquired from the Human Protein Atlas online database (Supplementary Figure 1C). Then, we also evaluated alteration frequency of TREM family gene and results revealed that amplification was the most common type of genetic mutation and the genetic alterations in TREM1, TREM2, TREML1, TREML2 and TREML4, which were approximately 6.0%, 6.0%, 9.0%, 8.0%and 8.0%, respectively (Supplementary Figure 1D). In terms of prognosis, survival analysis indicated a strong correlation between high expression of TREM family genes and poor clinical prognosis in OV patients (Figure 2(A-E)). The same result can be obtained from other GEO datasets based on Kaplan-Meier plotter website (Figure 2(F-K)).

### Correlation analysis between TREM1 expression and clinical characteristics

**Figure 3:**
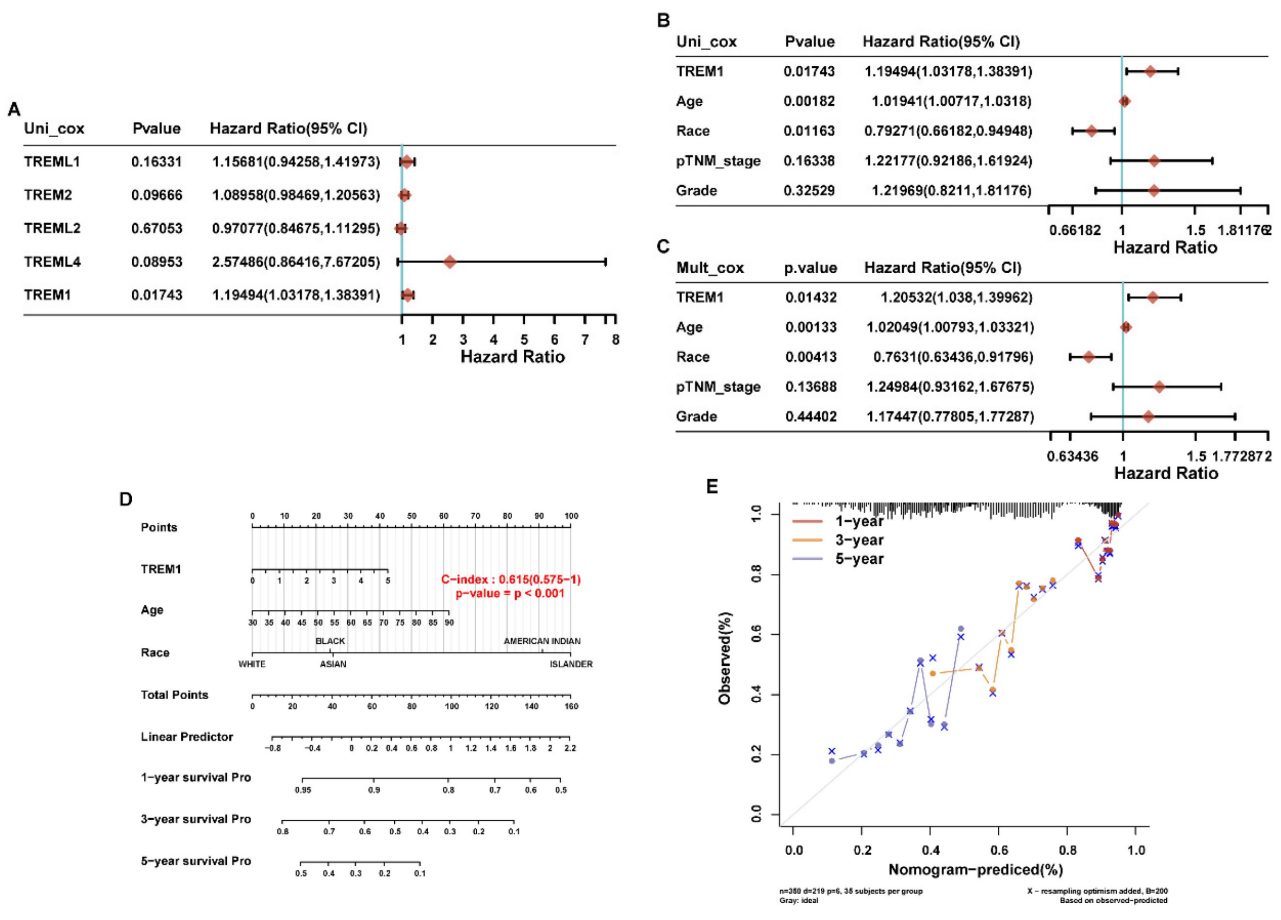
The Correlation analysis between TREM1 and prognostic predictors and clinical characteristic in ovarian cancer. (A) Univariate Cox analysis data of TREMs family members in TCGA cohort. (B-C) Univariate and multivariate analyses of the relation between the TREM1 and clinicopathological characteristics in the TCGA cohort. (D)Nomogram model for the probability of 1-, 3- and 5- years OS predictions. (E) Calibration curve of nomogram for OS. Abbreviations: OS, overall survival.

To further analyze the prognostic risk factor for OV patients, we firstly performed univariable Cox regression analysis on above-mentioned genes to identify the candidate genes with significant prognostic significance. Results revealed that TREM1 was the only gene showing a risk significance in OV patients (Figure 3A). Then, TREM1 and clinical features of ovarian cancer (age, race, TNM stage and grade) were evaluated in univariable and multivariable Cox analysis. The results showed that TREM1 expression (univariate hazard ratio (HR): 1.19, *p=*0.01743; multivariate HR: 1.21, *p=*0.01432), age (univariate HR: 1.02, *p=*0.00182; multivariate HR: 1.02,* p=*0.00133), race (univariate HR:0.79, *p=*0.01163; multivariate HR: 0.76, *p=*0.00413) in the TCGA-OV cohorts. All in all, TREM1 may be an independent prognostic biomarker for OV patients (Figure 3(B-C)). In addition, the significant clinical prognostic variables (age and race) identified in the study were performed to construct nomograms to estimate 1-, 3- and 5-year OS, respectively (Figure 3D). What is more, good consistency was established between nomogram survival probability and actual observable data using calibration curves (Figure 3E).

### Validation of the expression and prognosis of TREM1 in TMA specimens

**Figure 4:**
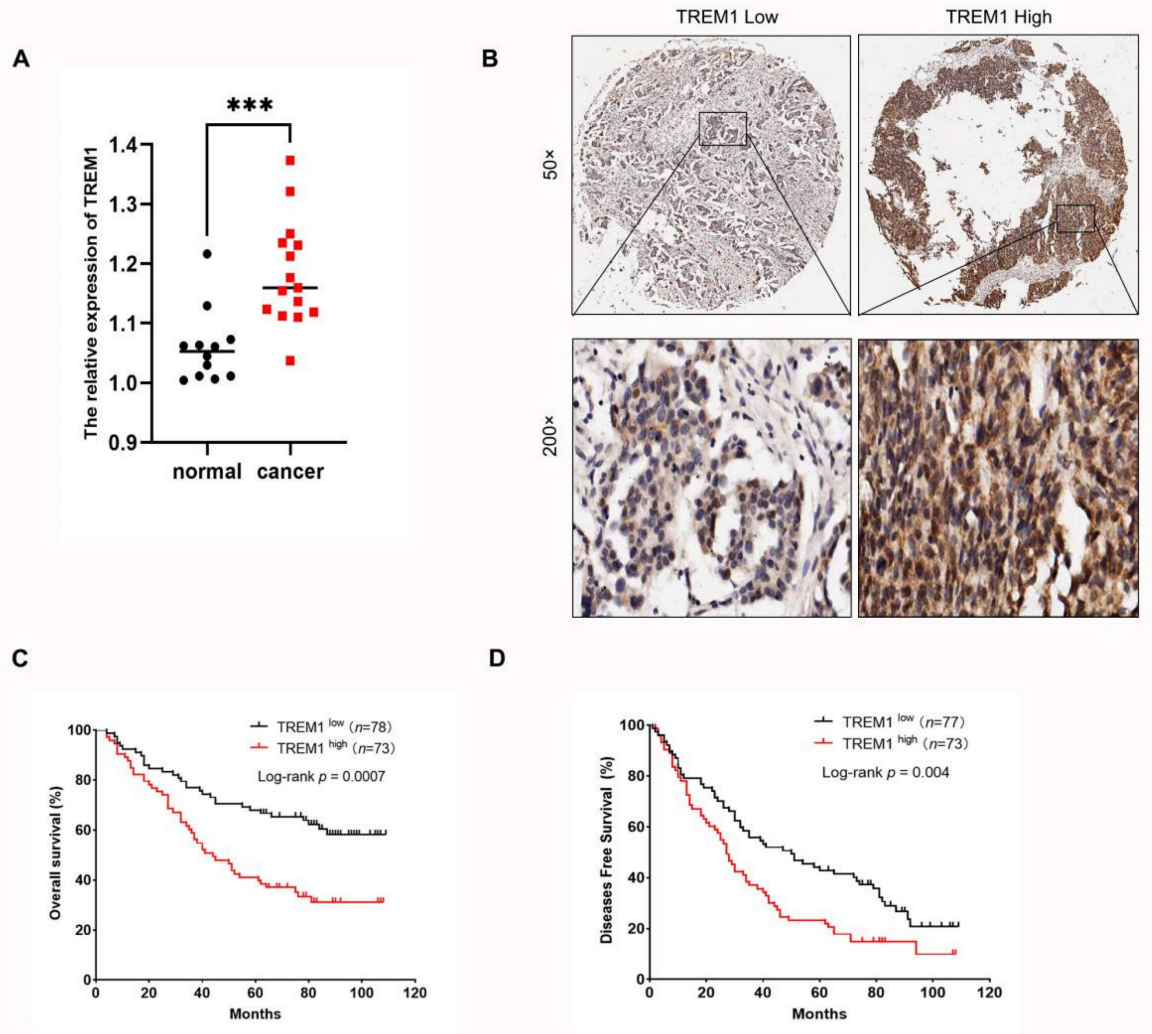
High expression of TREM1 in OV tissues predicts a poor clinical outcome. (A) The expression of TREM1 mRNA was determined in OV tissues and normal tissues. (B) Representative IHC images of TREM1 staining in OV tissues (magnification, 50X and 200X). (C) Kaplan-Meier analysis of the correlation between TREM1 expression and OS. (D) Kaplan-Meier analysis of the correlation between TREM1 expression and DFS disease-free survival. ***p < 0.001. Abbreviations: OS, overall survival; DFS, disease-free survival.

To further validate the relationship between TREM1 expression and prognosis of OV, we firstly performed RT-qPCR to compare the mRNA expression levels of TREM1 in tumor and normal tissues from clinical samples. The results showed that the expression of TREM1 was significantly high-expressed in tumor tissues (Figure 3A). Then, we also investigated the expression of TREM1 by IHC in an independent cohort of OV patients. Representative images of OV samples with low and high expression of TREM are shown in Figure 4B. Further analysis demonstrated that the TREM1 protein expression was significantly linked with histological differentiation, N, M-stages, and TNM-stage, while there was no significant association was observed with T stage or age (Table 1). Additionally, survival data also indicated that high expression group had a poorer OS (Figure 4C) and disease-free survival (DFS) (Figure 4D).

### Gene correlation and functional enrichment analysis of TREM1

**Figure 5:**
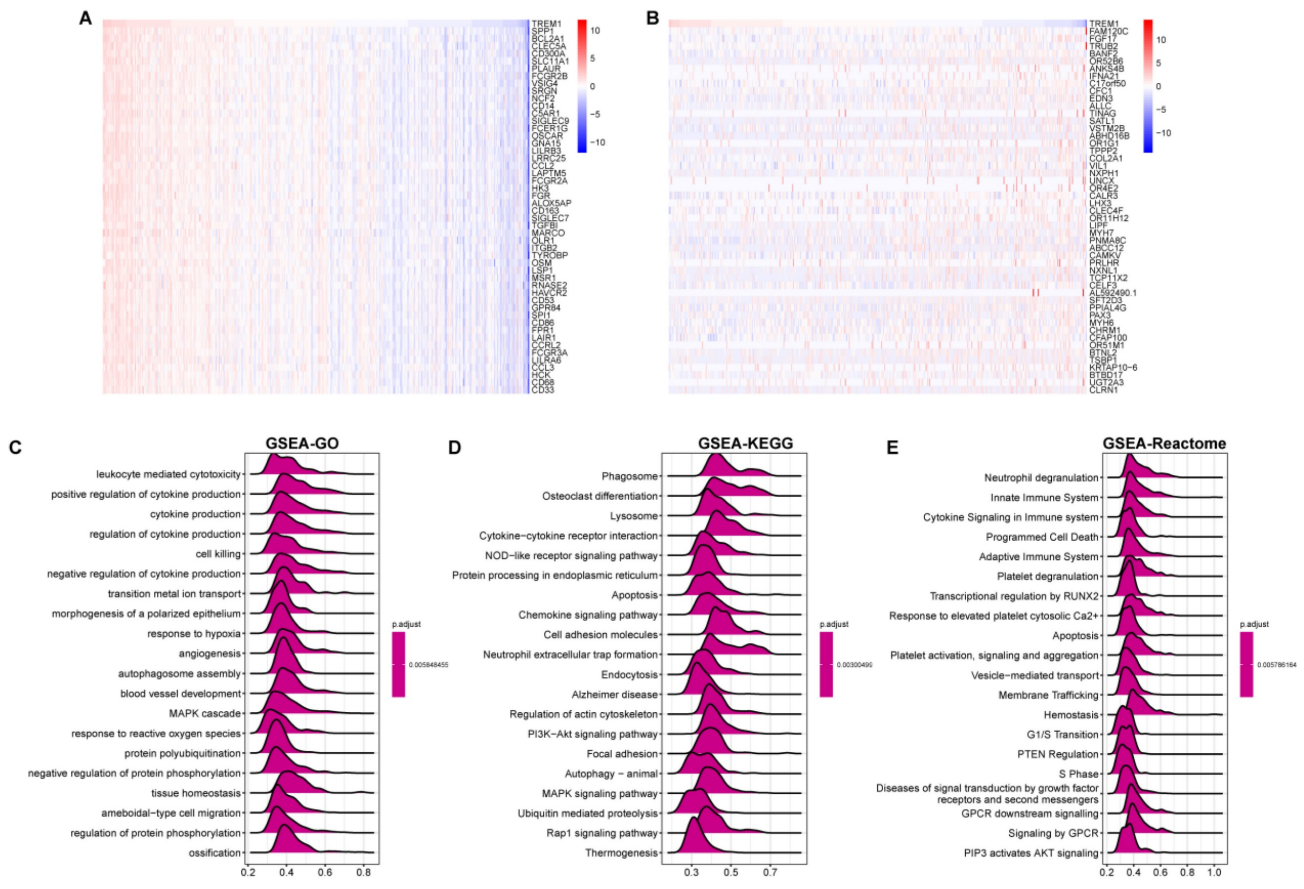
Gene correlation and functional enrichment analysis of TREM1. (A-B) Heat maps showing the top 50 significant genes positively and negatively correlated with TREM1 (C) GO enrichment analysis; (D) KEGG pathway enrichment analysis; (E) Reactome pathway analysis. Top 20 enriched pathways were exhibited.

To identify possible functionally related genes and potential regulatory patterns, we performed correlation analysis for TREM1 and all genes by using the TCGA-OV. The heatmap displayed the top 50 significantly positively and negatively linked genes with TREM1 (Figure 5 A-B). Next, we utilized gene set enrichment analysis (GSEA) to investigate TREM1 prospective roles by using GO, KEGG, and Reactome pathway. GO functional annotation indicated that these genes were significant enrichment for immune responses, including cytokine production, positive/ negative regulation of cytokine production, MAPK cascade, leukocyte mediated cytotoxicity (Figure 5C). *KEGG* pathway enrichment data revealed that these genes were predominately associated to immunological- and cancer-related pathways, specifically in cytokine signaling, cytokine-cytokine receptor interaction, NOD-like receptor signaling pathway and PI3K-Akt signaling pathway (Figure 5D). At the same time, *Reactome* pathway enrichment analyses also revealed that these genes were majorly related to immunological response, including neutrophil degranulation, immune system cytokine signaling, innate and adaptive immune systems (Figure 5E). Overall, these findings verified that TREM1 have a significant function in immune response and tumor development.

### Tumor microenvironment and immune cell infiltration analysis of TREM1

**Figure 6:**
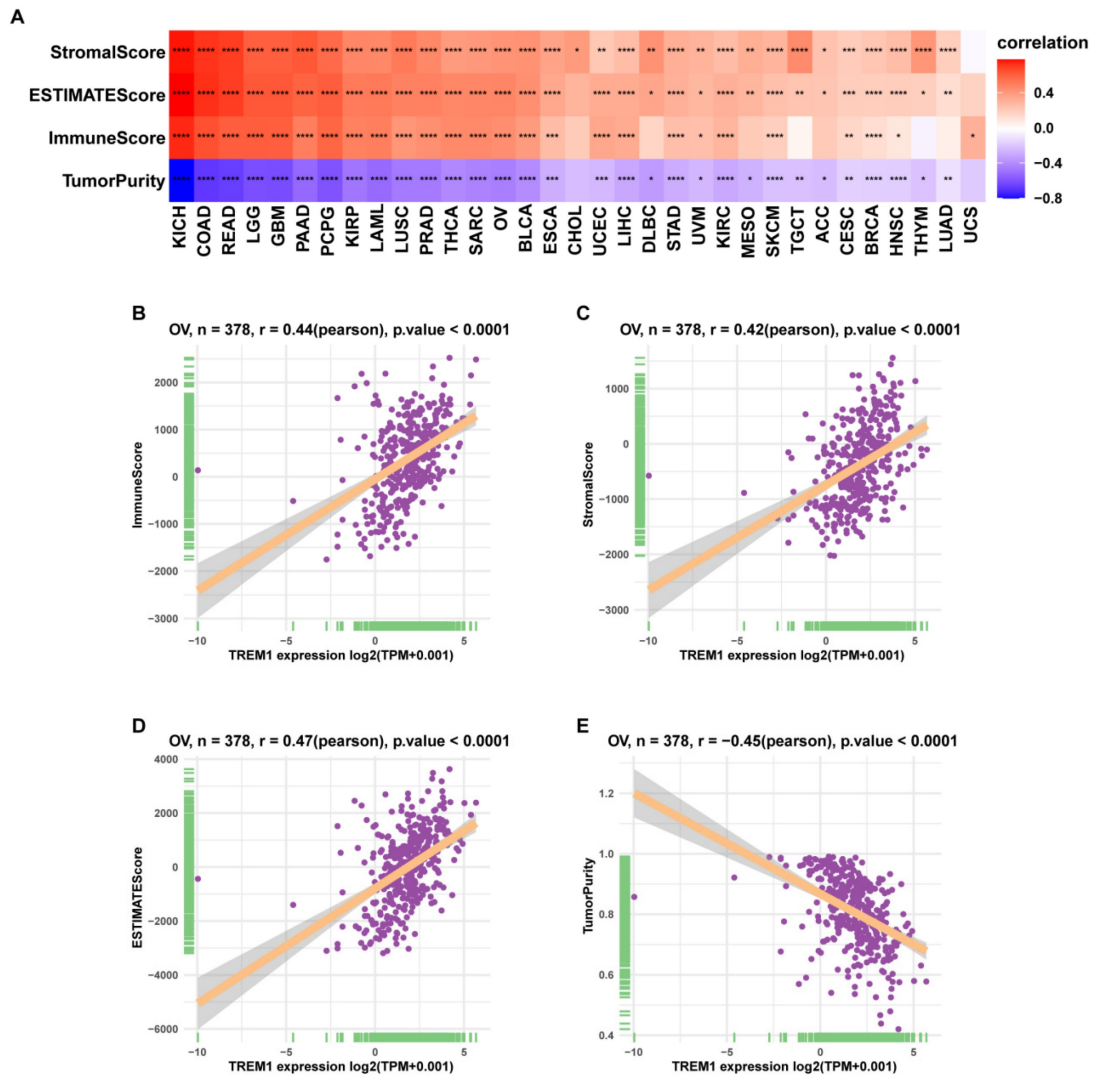
The correlation between TREM1 expression and tumor microenvironment. (A)Correlation between immune/stromal/estimate score and TREM1 expression in pan-cancer. (B-E) Correlation between stromal/immune/estimate scores and tumor purity and in the TCGA-OV cohort. *p < 0.05, **p < 0.01, ***p < 0.001, and ****p < 0.0001.

**Figure 7:**
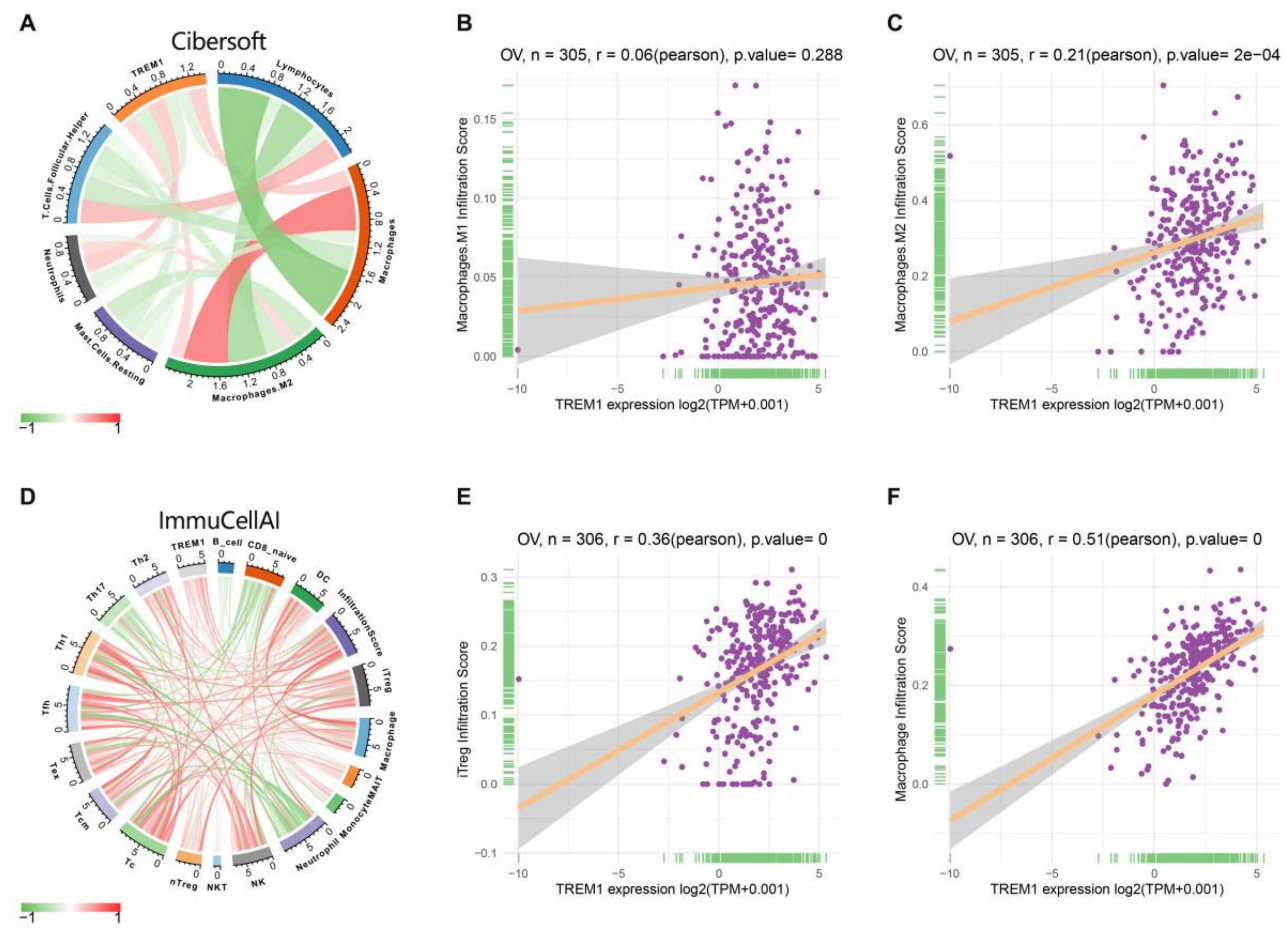
The correlation between TREM1 expression and tumor infiltration of different immune cells. (A) Correlations between TREM1 expression and immune cell infiltration from published data. (B-C) Correlation between TREM1 expression and macrophages infiltration. (D) Correlations between TREM1 expression and immune cell infiltration from ImmuCellAI database. (E-F) Correlation between TREM1 expression and macrophages and iTregs infiltration. Abbreviations: iTregs, induced regulatory T cells.

Since the above results confirmed that TREM1 has a necessarily close connection with multiple immune molecules and pathways, we then evaluated connection between TREM1 expression and tumor microenvironment (TME). As known TME is mainly comprised of tumor cells, immunological and stromal cellular components, through the ESTIMATE algorithm, the ratio of the immune matrix components of each sample embodied in ImmuneScore and StromalScore. As shown in Figure 6A, the clustering revealed that TREM1 expression had a significant connection with the immune score and the stromal score in pan-cancer analysis. Consistently, the same results were also observed in OV (Figure 6(B-D)). After confirming the internal association between TREM1 expression and immune microenvironmental value, we further explored the relationship between TREM1 and immunological cell types infiltration. Data from TIMER2 database revealed that TREM1 expression was positively correlated with CD4+ T Cell, macrophage, dendritic cell, neutrophil and negatively related to B cells (Supplementary Figure 2A). Then, through the CIBERSORT algorithm, we investigated the correlation between TREM1 expression and the proportions of 26 immune cell subsets according to published data. The correlation circle plot showed that macrophage, neutrophil infiltration were significantly positively correlated with TREM1 expression, however negatively correlated with CD8+ T cells (Figure 7A). It was remarkable that TREM1 expression had varied relationships with distinct subsets of infiltrating macrophages, which significant associated to infiltrating M2 macrophages but not M1 macrophages (Figure 7(B-C)). Results from multivariate Cox proportional hazards model are shown that high TREM1 expression with high M2 macrophages infiltrating predicts a worse prognosis (Supplementary Figure 2B). Additionally, considering about that immune infiltration level were significantly correlated with development in tumors, we also used ImmuCellAI database to further (Figure 7D). support our findings (Figure 7D). In line with above results, TREM1 was positively associated with macrophage and regulatory T cells (Tregs) (Figure 7(E-F)). In general, these findings indicate that TREM1 may act as a tumor suppressor in the immune microenvironment, which increase the infiltration levels of tumor-associated macrophages (TAMs) and Tregs and induce immune-suppression state. This could partly explain why high TREM1 expression are linked with worse prognosis in OV.

### Pan-cancer analysis of TREM1 in human cancers

**Figure 8:**
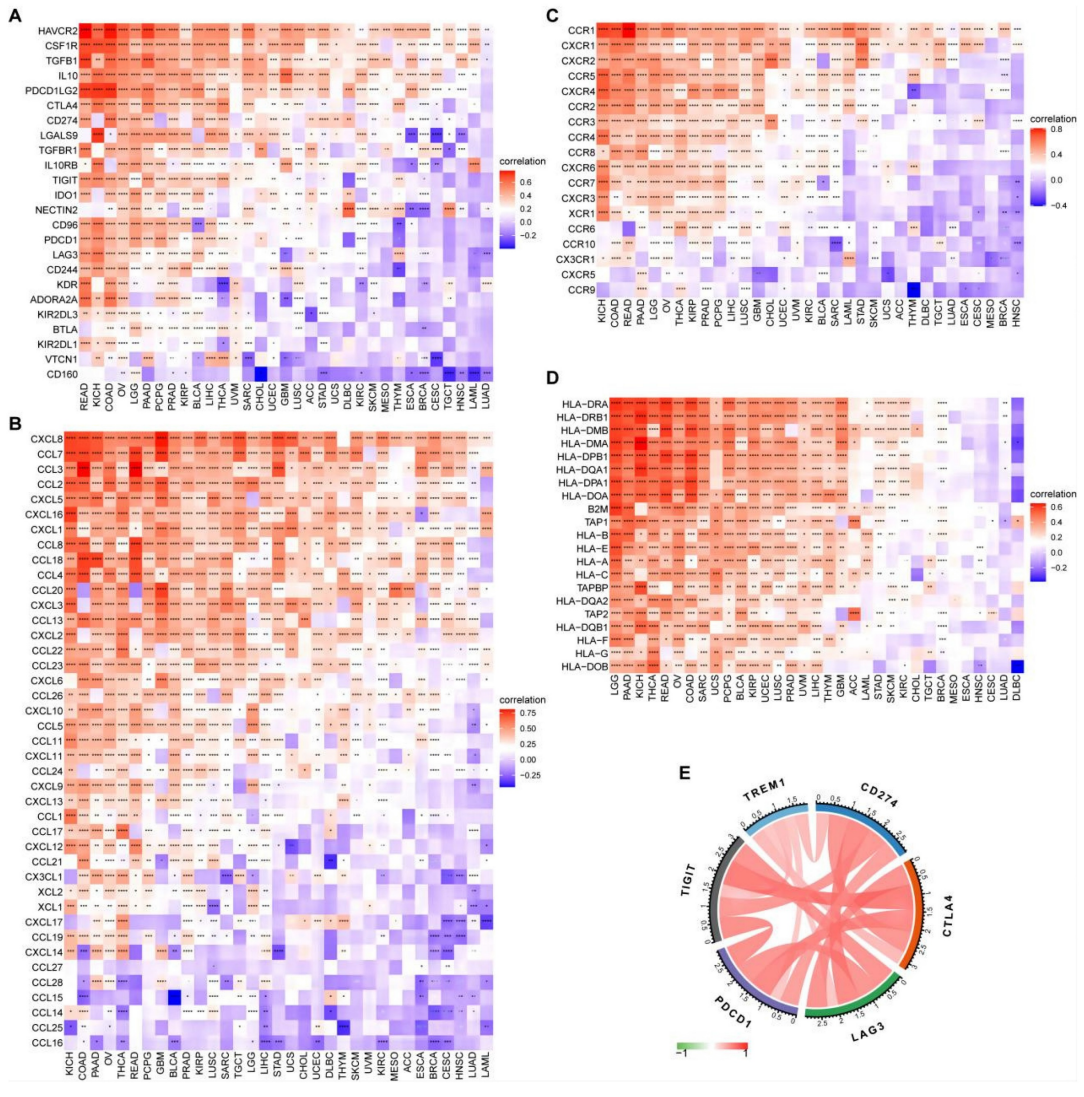
The correlation between TREM1 expression and immune-associated genes. (A-D) Correlation between TREM1 and immunosuppressive genes, chemokines gene, chemokine receptors gene and MHC genes in pan-cancer. (E) Correlation between TREM1 expression and immune checkpoint genes expression in OV. *p < 0.05, **p < 0.01, ***p < 0.001, and ****p < 0.0001.

Considering the lack of TREM1-related cancer research, we explored the relevance of ^18, 21^our findings in 34 types of human common cancer from the TIMER2 database. Collectively, TREM1 was overexpressed in multiple cancer types (Supplementary Figure 3A). Integrated data from TCGA and GTEx databases also indicated that highly abnormal changes of TREM1 expression in normal and cancer tissue (Supplementary Figure 3B). After identifications the relationship between TREM1 expression and the immune microenvironment, we performed gene co-expression studies to determine the relationships between TREM1 expression and immune-related genes. The heat maps illustrated that TREM1 expression was strongly associated with immunosuppressive genes (Figure 8A), chemokines (Figure 8B), chemokine receptors (Figure 8C) and MHC genes (Figure 8D) across most tumor types, except lung adenocarcinoma (LUAD), acute myeloid leukemia (LAML), head and neck squamous cell carcinoma (HNSC), cervical squamous cell carcinoma as well as endocervical adenocarcinoma (CESC). In addition, immune checkpoints are widely known to help tumor cells in evading immune surveillance and to affect patient's prognosis. We next estimate the link between TREM1 expression and immune checkpoints in pan-cancer, and a strong correlation was revealed in multiple types of cancers (Supplementary Figure 3C). Notably, we found that some commonly immune checkpoints, including PDL1, LAG3, CTLA4, CD276, PDCD1 and TIGIT, are strongly related to TREM1 expression in OV (Figure 8E). Furthermore, correlation analyses between TREM1 and tumor mutational burden (TMB) and microsatellite instability (MSI) in pan-cancer were conducted (Supplementary Figure 3E). Radar chart showing the connection between TREM1 expression and TMB was significant in eight cancer types, and the correlation with MSI was also reflected in four types of cancer. Finally, univariate Cox regression analyses were utilized to elucidate correlation of TREM1 expression with OS and progression-free survival (PFS) of patients in TCGA pan-cancer. The OS results indicated that TREM1 is a risk factor for patients with glioblastoma multiforme (GBM), kidney renal clear cell carcinoma (KIRC), lower grade glioma (LGG), liver hepatocellular carcinoma (LICH), mesothelioma (MESO), pancreatic adenocarcinoma (PAAD) and stomach adenocarcinoma (STAD). The PFS analysis revealed that TREM1 acted as a risk factor for patients with breast invasive carcinoma (BRCA), KIRC and LGG and a protective factor for patients with lymphoid neoplasm diffuse large B-cell lymphoma (DLBC) (Supplementary Figure 3D).

## Discussion

Over the past decade, tumor immunotherapy has dramatically changed the treatment paradigm for patients with multiple cancers, which is rapidly evolving as one of the major pillars of cancer treatment. Among the many immunotherapeutic strategies, immune checkpoint blockade therapy has significantly improved the treatment prospects of patients with melanoma, non-small-cell lung cancer (NSCLC), renal cell carcinomas (RCC) and other cancers^22^-^26^. However, the survival benefits of ICIs (immune checkpoint inhibitors) treatment in clinical trials for OV patients have not been significant^27^. In the KEYNOTE-028 clinical trial, 26 patients were enrolled in the ovarian cancer cohort. The objective response rate (ORR) to pembrolizumab was 13%, with a progression-free survival (PFS) of only 1.9 months and an overall survival (OS) of 13.1 months^28^. The poor efficacy of ICIs in treating OV is currently attributed to the fact that OV is considered an immunologically “cold” tumor. Factors such as immune suppression and insufficient antigen presentation capability within the tumor microenvironment limit the effectiveness of ICIs treatment^29^, ^30^. To improve the rate of response to ICB therapies, continuous efforts should be taken into uncovering the mechanisms of tumor immune evasion and development of more sensitive and reliable biomarkers for diagnosis or prognosis of OV.

Study of relevant literature show that TREM1 not only play vital roles in innate and adaptive immunity but also closely linked to malignant diseases^31^. For example, hypoxia-induced upregulation of the expression of TREM1 in macrophages increases the release of CSF-1, which promotes invasion and vascular mimicry in GBM cells^32^. TREM1 promotes resistance to arginine-deprivation therapy by activating the AKT/mTOR/STAT3 signaling pathway and upregulating its downstream effector CCL2 in breast cancer^33^. In prostate cancer (PCa), upregulation of the TREM-1 mediated the activation of androgen receptor signaling in macrophages to promote PCa-derived cancer cell progression^34^. Furthermore, survival analysis has shown that high expression of TREM1 was related to poor overall survival (OS) and disease-free survival (DFS) in NSCLC, Hepatocellular carcinoma (HCC) and Renal cell carcinoma^11^, ^35^, ^36^. In fact, preclinical studies have demonstrated that blocking TREM1 have positive effects in cancer therapy. A variety of strategies have been developed to inhibit TREM1 signaling^21^, ^37^, ^38^. In this article, we investigated TREM1 expression patters in OV, and found TREM1 expression was significantly higher in OV tissues. Then, prognostic and clinical association analyses demonstrated that high TREM1 expression predicted poor OS and DFS for OV patients. In brief, these findings suggest that TREM1 could be a potential biomarker for clinical treatment.

Next, to investigate the potential biological mechanism of TREM1 in OV, GSEA revealed that TREM1 was associated with multiple oncogenic pathways (e.g. MAPK, PI3K-Akt, angiogenesis, cell cycle and apoptosis). Another more important discovery is that TREM1 was intimately associated with immune-related pathways, such as adaptive immune system and innate immune system, neutrophil degranulation and cytokine signaling pathways. Further investigation on ESTIMATE scores analysis implied that TREM1 expression showed positive correlation positive correlations with both stromal and immune cell content in the TME, indicating that the tumor-infiltrating immune cells (TIICs) might contribute to tumor progression. Therefore, we hypothesized that the pathways modulated TREM1 could affect the immune microenvironment of the ovarian tumors and thus influencing the biological behavior of carcinomas. Interestingly, current research has shown that TREM1 is expressed on tumor-associated macrophages (TAMs), dendritic cells (DCs), cancer-associated fibroblasts (CAFs) and tumor-infiltrating neutrophils within the TME. Such a TREM1^+^ TME facilitates tumor cells to acquire hallmark capabilities of cancer including sustained proliferative signaling, invasion, metastasis and immune evasion. In mouse melanoma (B16F10) and fibrosarcoma (MCA205) models, genetic or pharmacological inhibition of TREM1 expression could reduce the infiltration of myeloid derived suppressor cells (MDSCs), while increasing the proportion of cytotoxic CD8+T to inhibit tumor growth^21^. In hepatocellular carcinoma cells (HCC), Wu *et al.* found that TREM-1+ TAMs are abundant in the hypoxic environment of HCC. By indirectly reducing CD8+ T cell cytotoxicity and causing CD8+ T cells apoptosis eventually contribute to tumor immune suppression and progression. Interestingly, programmed cell death ligand 1 (PD-L1) blockade failed to reverse TREM-1+ TAMs-mediated immunosuppression as TREM1-induced upregulation of CCL20 and promoted the aggregation of Foxp3+regulatory T cells (Tregs)^16^. High expression of TREM1 in papillary thyroid cancer (PTC) could promote an immunosuppressive microenvironment by enhancing Treg infiltration and decreased CD8+T cells infiltration^17^. Here we performed a comprehensive analysis of the relation between TREM1 expression patterns and tumor microenvironments in OV and found that TREM1 was significantly positively correlated with Tregs and macrophage M2. We speculated that the expression of TREM1 in OV may promote tumor progression by altering the phenotype of TAMs. After all, in the setting of the TME in OV, TAMs generally exhibit an immunosuppressive M2 phenotype, which promote tumor progression by several mechanisms that include the secretion of immune-suppressive factors and pro-angiogenic factors, reduction the activity of effector lymphocytes and the promotion of Treg development^39^. Studies showed that TAMs overall confer a poor prognosis in ovarian cancer^40^-^42^. In the present study, CIBERSORT were used to infer immune cell infiltrations, suggesting that TREM1 was significantly positively correlated with Tregs and macrophage M2. In addition, pan-cancer analysis showed that cytokines (IL-10, TGF-β and CSF-1) and chemokines/ chemokine receptors (CCL18, CCL12, CCL22 and CCR2) participating in immunosuppressive process and co-inhibitory checkpoints (CTLA-4, LAG-3, TIGIT and PDCD1) were all positively correlated with expression of TREM1. Therefore, TREM1 may participate in the antitumor immunity by regulating immune-related cytokine and immunosuppressive cell in TME, suggesting that targeted TREM1 to inhibit its expression may provide potential way for improving treatment effectiveness of immunotherapy.

Nevertheless, this study has some significant limitations that must be acknowledged. Firstly, we obtained the conclusions mostly through bioinformatics and public databases, the specific expression level of TREM1 and its role in ovarian cancer remains to be studied. Secondly, while we revealed a close connection between TREM1 expression and immune microenvironment, the detailed regulatory mechanism to explain these observations requires more basic experiment to systematically elucidated in the future.

## Conclusion

In summary, our observations demonstrated that TREM1 is upregulated in ovarian cancer, and its elevated expression level is related to poor prognosis and clinical characteristics. Most importantly, immune cell infiltration analysis revealed that TREM1 expression was closely linked to the infiltration of Tregs and tumor-associated macrophages, which may contribute to create a tumor immunosuppressive microenvironment and favor tumor progression. Therefore, TREM1 may serve as a positive prognostic marker for OV and its potentiality in predicting immunotherapy response.

## Supplementary Material

Supplementary figures and tables.

## Figures and Tables

**Table 1:** Correlations between TREM1 expression and clinicopathologic parameters in patients with ovarian cancer

	Clinicopathological features	TREM1 expression		Total	P
		High	Low		
Age(years)					0.916
	<50	37	34	71	
	>=50	41	39	80	
Grade					0.001
	I	6	10	16	
	II	15	3	18	
	III	31	54	85	
T stage					0.145
	I	7	2	9	
	II	21	16	37	
	III	49	55	104	
N stage					0.002
	N0	65	45	110	
	N1	12	28	40	
M stage					
	M0	68	50	118	0.003
	M1	9	23	32	
TMN stage					0.016
	I	7	2	9	
	II	21	16	37	
	III	40	32	72	
	IV	9	23	32	
