# Supplementary material for: Expressional and prognostic value of TREM1 in ovarian cancer: A comprehensive study based on bioinformatics and clinical analysis validation
Source: J Cancer. 2025 Jan 1;16(2):577–89. doi: 10.7150/jca.101152 (PMC11685700; doi:10.7150/jca.101152)
Supplement: Supplementary file 1 — Supplementary figures and tables. [file jcav16p0577s1.zip › Supplementary Figures.doc]

Supplementary Fig. 1 Analysis of the TREMs family genes. (A) Gene expression correlation analysis of TREMs family genes in TCGA-OV cohort. (B)The STRING database for analysis of protein-protein interaction (PPI) network of TREMs family genes. (C)The HPA database for analysis of representative immunohistochemical images of protein expression of TREMs in cancer and normal tissues. (D)The cBioPortal analysis for analysis of genetic alterations of TREMs family genes in ovarian cancer.

Supplementary Fig. 2 The Correlation analysis between TREM1 and immune infiltrate populations. (A) The correlation of TREM1 expression and six types of immune infiltrates populations from TIMER2 database. (B) The correlation of TREM1 and M2 macrophages expression and the survival rate of OV patients from TIMER2 database.

Supplementary Fig. 3 Overview of TREM1 in human cancers. (A) Pan-cancer expression profile of TREM1 from TIMER2 database (B) Pan-cancer expression profile of TREM1 from TCGA and the GTEx databases. (C) The correlations between TREM1 expression and immune-checkpoint-related gene expression. (D)The correlations between TREM1 expression and survival analysis. (E) The relationship of TREM1 expression and TMB, MSI in OV.

Abbreviations: OS, overall survival; FPS, progression free survival; TMB, tumor mutational burden; MSI, microsatellite instability;
